# Normalized periprostatic fat MRI measurements can predict prostate cancer aggressiveness in men undergoing radical prostatectomy for clinically localised disease

**DOI:** 10.1038/s41598-017-04951-8

**Published:** 2017-07-05

**Authors:** Naief Dahran, Magdalena Szewczyk-Bieda, Cheng Wei, Sarah Vinnicombe, Ghulam Nabi

**Affiliations:** 1 Division of Cancer Research, School of Medicine, University of Dundee, Dundee, DD1 9SY UK; 2 Department of Anatomy, Faculty of Medicine, University of Jeddah, Jeddah, Saudi Arabia

## Abstract

Periprostatic and pelvic fat have been shown to influence prostate cancer behaviour through the secretion of chemokines and growth factors, acting in a paracrine mode. We have measured periprostatic fat volume (PFV) with normalisation to prostate gland volume on pelvic magnetic resonance imaging (MRI) and have correlated this with grade (Gleason score; GS) and pathological staging (pT) of prostate cancer (PCa) following radical prostatectomy (RP). PFV was determined using a segmentation technique on contiguous T_1_-weighted axial MRI slices from the level of the prostate base to the apex. The abdominal fat area (AFA) and subcutaneous fat thickness (SFT) were measured using T_1_-weighted axial slices at the level of the umbilicus and the upper border of the symphysis pubis, respectively. PFV was normalised to prostate volume (PV) to account for variations in PV (NPFV = PFV/PV). Patients were stratified into three risk groups according to post-operative GS: ≤6, 7(3 + 4), and ≥7(4 + 3). NPFV was significantly different between the groups (*p* = 0.001) and positively correlated with post-operative GS (ρ = 0.294, *p* < 0.001). There was a difference in NPFV between those with upgrading of GS from 6 post prostatectomy (2.43 ± 0.98; *n* = 26) compared to those who continued to be low grade (1.99 ± 0.82; *n* = 17); however, this did not reach statistical significance (*p* = 0.11).

## Introduction

Several reports have found an association between obesity and aggressivity of prostate cancer (PCa) including an increased risk of biochemical recurrence following radical prostatectomy (RP)^1–3^. Most of these studies used body mass index (BMI) (kg/m2), which is a marker of generalised obesity. The calculation of BMI is based on the whole body weight and height. However, the BMI may not reflect the amount of metabolically active visceral and specifically, periprostatic and pelvic cavity fat. A number of factors, such as status of angiogenesis, adipogenesis, genetic regulation and insulin resistance determine the distribution of metabolically active fat in an individual^4^.

Periprostatic fat contains highly active adipocytes, which act as paracrine cells with the ability to secrete many growth factors, chemokines and inflammation-modifying molecules^4, 5^. Body fat is known to produce Interleukin-6 (IL-6), tumour necrosis factor-α (TNFα), leptin, and adiponectin, which have been shown to influence prostate cancer progression^6–9^. Free fatty acids (FFA), which are released from adipocytes closely spatially related to cancers are found to be the major source of energy for tumour cells in a number of cancers including prostate cancer^10, 11^.

There are several indications that periprostatic fat abundance can influence prostate cancer aggressiveness through paracrine effects^12, 13^. Inhibition of some of chemokine pathways, in particular CCR3/CCL7, prevents local dissemination of prostate cancer^12^. The CCR3/CCL7 chemokine pathway may promote extraprostatic extension of aggressive prostate cancers into periprostatic fat, and modification of the phenotype and characteristics of adipose cells occur which are then termed “cancer-associated adipocytes”^12, 14^.

Measurement of periprostatic fat as a marker of prostate cancer aggressiveness has been reported using computed tomography (CT) in men opting for prostate brachytherapy or external beam radiation, with conflicting results^7, 8^. Woo *et al*.^15^, in a retrospective review of MRI performed before radical prostatectomy (RP), showed a significant correlation between Gleason score (GS) of prostate cancer in prostatectomy specimen and periprostatic fat thickness^15^. Similarly, Zhang *et al*.^16^, in 184 men undergoing RP found a positive correlation between periprostatic fat area and prostate cancer aggressiveness^16^. Both reports, however, used different methods of periprostatic fat measurement. Woo *et al*. used the shortest perpendicular distance from pubic symphysis to prostate, whereas Zhang *et al*. measured periprostatic fat area, including ischiorectal fossae measurements. Also, a single transverse slice at the level of the femoral head and greater trochanter of the femur was used to give a measure of area instead of total volume. In a more recent study, Tan *et al*.^17^ found that periprostatic fat volume correlated with GS of prostate cancers on transrectal ultrasound guided biopsies^17^. This study did not use histopathology of RP as a reference standard and thus has limitations. Table 1 summarises various techniques, imaging modalities and methods used for estimations of periprostatic fat. Clearly, various reference standards and comparative measures have been used in the literature and this may have influenced the outcomes of these studies.

**Table 1:** Periprostatic fat measurement techniques on imaging and their outcomes in previous studies.

Study	Reference standard	Treatment	Method	Imaging modality	Outcome
**Woo** ***et al***.^15^ Korea *N* = 190	Radical prostatectomy	Surgery	Periprostatic and subcutaneous fat thicknesses.	Preoperative mid-sagittal T_2_W MRI. Measured the shortest perpendicular distance from symphysis pubis to skin and prostate.	Positive correlation between the periprostatic fat thickness and GS^*^
**Roermund** ***et al***.^7^ Netherlands *N* = 902	Prostate biopsies (? number)	Brachytherapy	Periprostatic fat area (cm^2^)/subcutaneous fat thickness (cm).	Two transverse 3 mm-thick CT slices at different levels. Pelvic fat was defined by the density range −190 to −30 HU^*^. Fat density (%) was calculated by dividing periprostatic fat area by total contour area.	Periprostatic fat area and density were not correlated with Prostate cancer aggressiveness
**Roermund** ***et al***. **(2010)** Netherlands *N* = 932	Prostate biopsies (? number)	External radiotherapy and brachytherapy	Periprostatic fat area (cm^2^)/Subcutaneous fat thickness.	Single transverse CT slice at the level of the femoral head and greater trochanter. Defined the pelvic fat by the density range −190 to −30 HU^*^. Fat density (%) was calculated by dividing periprostatic fat area by total contour area.	Higher periprostatic fat density had more often aggressive prostate cancer.
**Bhindi** ***et al***.^25^ Canada *N* = 931	10–12 prostate biopsies	unknown	Periprostatic fat thickness.	Trans rectal ultrasonography (TRUS). The periprostatic fat was measured from the shortest perpendicular distance between pubic bone and prostate.	Periprostatic fat can be used as predictor for prostate cancer and high-grade prostate cancer at biopsy.
**Tan** ***et al***.^17^ USA *N* = 295	12 core prostate biopsies	unknown	Periprostatic fat volume and prostate volume	3T MRIs. Periprostatic fat was marked from the level of its base to apex on T_2_W MR images ‘’cranial to caudal”. Ratio was calculated (fat volume/prostate volume)	Higher ratio may be a risk factor, is significantly associated with higher Gleason score
**Zhang** ***et al***.^16^ China *N* = 184	Radical prostatectomy	Surgery	Periprostatic fat area (cm^2^) and Subcutaneous fat thickness	T_2_W transverse MRI slice at the level of the femoral head and greater trochanter of the femur.	Periprostatic fat can predict the prognosis of patient with radical retropubic prostatectomy.

*GS = Gleason score. *HU = Hounsfield unit.

**Figure 1:**
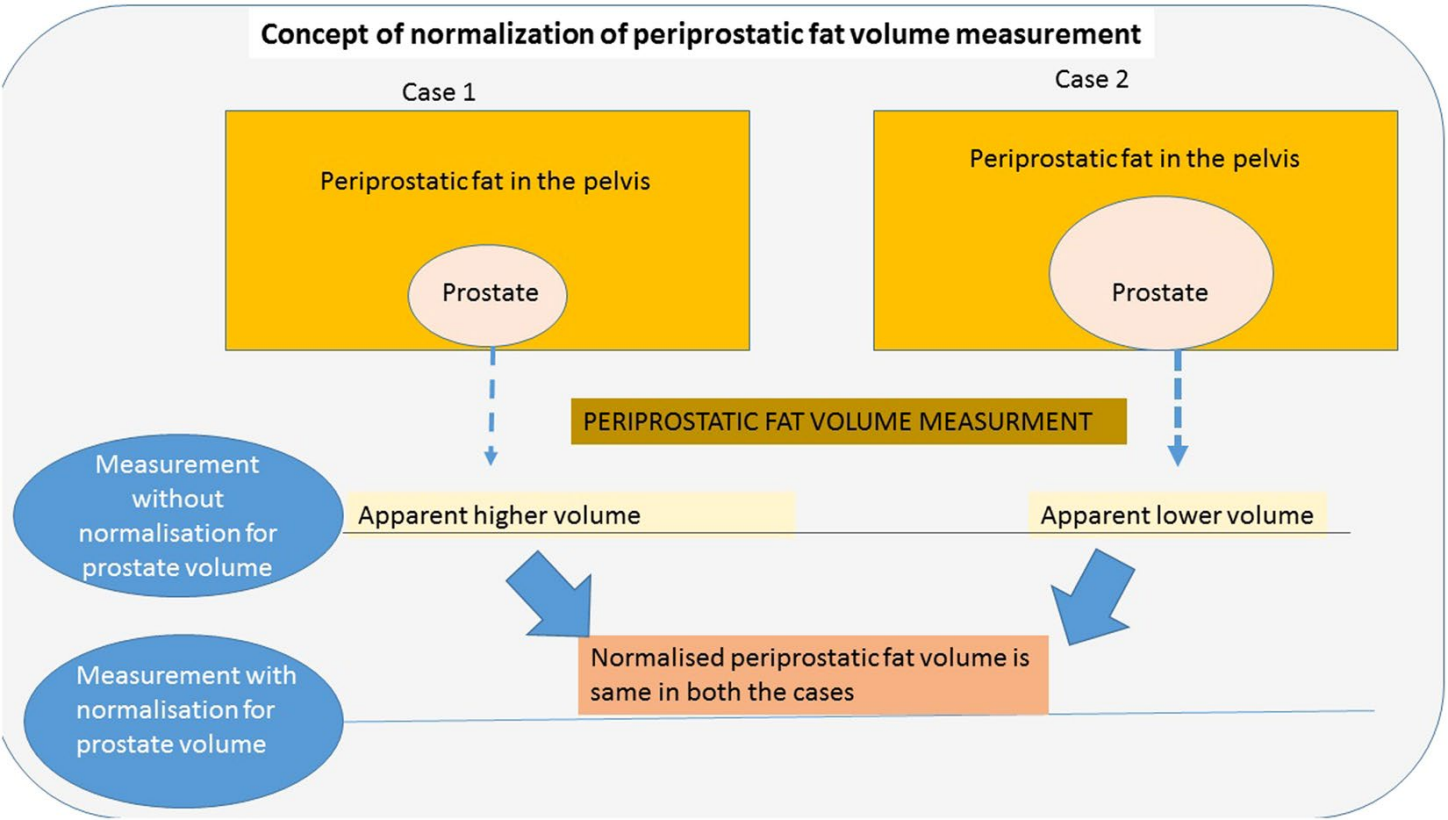
Schematic diagram explaining the concept of Normalised Periprostatic Fat Volume.

We hypothesised that normalisation of periprostatic fat volume to prostate size would provide a better reflection of relative fat volume in the pelvis and periprostatic area. The concept of normalisation is shown in Fig. 1. Therefore, the objectives of the present study were: 1. To measure periprostatic fat volume, subcutaneous fat thickness, and intra-abdominal fat area on anatomical MRI scans using a standardised measurement method. 2. To correlate fat measurements with grade (GS) and pathological staging (pT) of prostate cancer following RP.

## Patients and Methods

### Cohort Selection and Power Calculation

This is a prospective study with Caldicott institutional approval (Caldicott/CSAppGN021211). All experiments including the study protocol study followed approved institutional guidelines. The study had ethical approval (ethical approval number 13/ES/0099) with each participant informed consenting to the use of their imaging data. A sample size of 150 was calculated based on 5% significance level with an effect size of 0.3 and 80% power. Allowing for 8% drop out for poor data quality, we planned recruitment of a cohort of 162 men. Between January 2010 and December 2015, 162 men with localised prostate cancer opting for RP were recruited. Prospective data were acquired for baseline demographic characteristics, disease specific parameters and imaging including MRI of the prostate. Patient specific data were acquired through access to standard NHS online clinical databases. Study inclusion criteria were:

- Men with localised prostate cancer without prior radiotherapy or hormonal treatment.
- Radical prostatectomy as a treatment option.
- Gleason score and histopathological stage of RP specimens review by an experienced uropathologist and MRI review by uroradiologists.

Exclusion criteria were: men with metastatic prostate cancer or those with localised prostate cancer opting for external beam radiotherapy or brachytherapy.

### Imaging Protocol

MRI scans were acquired using either 3-T (*n* = 65) or 1.5-T (*n* = 97) Siemens MR units with pelvic phased array coils. All patients received 20 mg of butyl scopolamine (Buscopan; Boehringer-Ingelheim) injected intramuscularly before MRI to minimize peristalsis.

Axial, sagittal and coronal T2-weighted spin-echo sequences, axial T1-weighted spin-echo sequences and axial fat-suppressed single-shot echo-planar diffusion weighted imaging using a small field of view (SFV) of the pelvis were routinely obtained for all patients, according to the standard prostate cancer characterisation MRI protocol in use at the time. Dynamic contrast-enhanced imaging was performed on a subgroup of patients undergoing imaging at 3 T unit as part of the standard multiparametric prostate MRI protocol at our institution. The data obtained form a part of the ongoing MULTIPROS study (http://www.hra.nhs.uk/news/research-summaries/multipros-study/). Table 2 summarises the imaging protocol of all sequences used in this study.

**Table 2:** Imaging protocol.

		TR/TE (ms)	Slice thickness (mm)	Interslice gap (mm)	Matrix	FoV read (mm)	Parallel imaging acceleration factor
**1.5 Tesla MRI**							
T_1_W	Axial pelvic	650/11	3	0.6	320 × 320	200	2
	Trans. Pelvic LN	532/9.4	6	0.6	320 × 240	380	2
	Trans. Abdomen	166/4.76	6	0.6	256 × 192	380	2
T_2_W	Axial pelvic	3,890/100	3	0.6	512 × 512	200	2
**3.0 Tesla MRI**							
T_1_W	Axial pelvic	650/12	3	0.6	320 × 320	200	2
	Trans. Pelvic LN	755/13	6	0.6	320 × 240	280	4
	Trans. Abdomen	140/2.46	5	0.6	320 × 250	380	2
T_2_W	Axial pelvic	4,000/95	3	0.6	320 × 320	280	2

### Fat Measurements

After anonymization of MRI studies from 162 men who subsequently underwent RP for clinically non-metastatic disease, the PFV was determined by using a semi-automated segmentation technique on contiguous 3 mm T_1_-weighted axial slices. OsiriX MD © Pixmeo Sarl 2015 (http://www.osirix-viewer.com) software was used for these measurements. The software utilises bicubic interpolation to deal with interslice gaps (in this study, 0.6 mm for both T_1_ and T_2_-weighted sequences). It was established that segmentation was most accurate and reproducible on non fat suppressed T_1_-weighted images, which provided optimal contrast between adipose and all other non-adipose tissues. One observer carried out all segmentations and measurements, blinded to GS and postoperative T stage.

**Figure 2:**
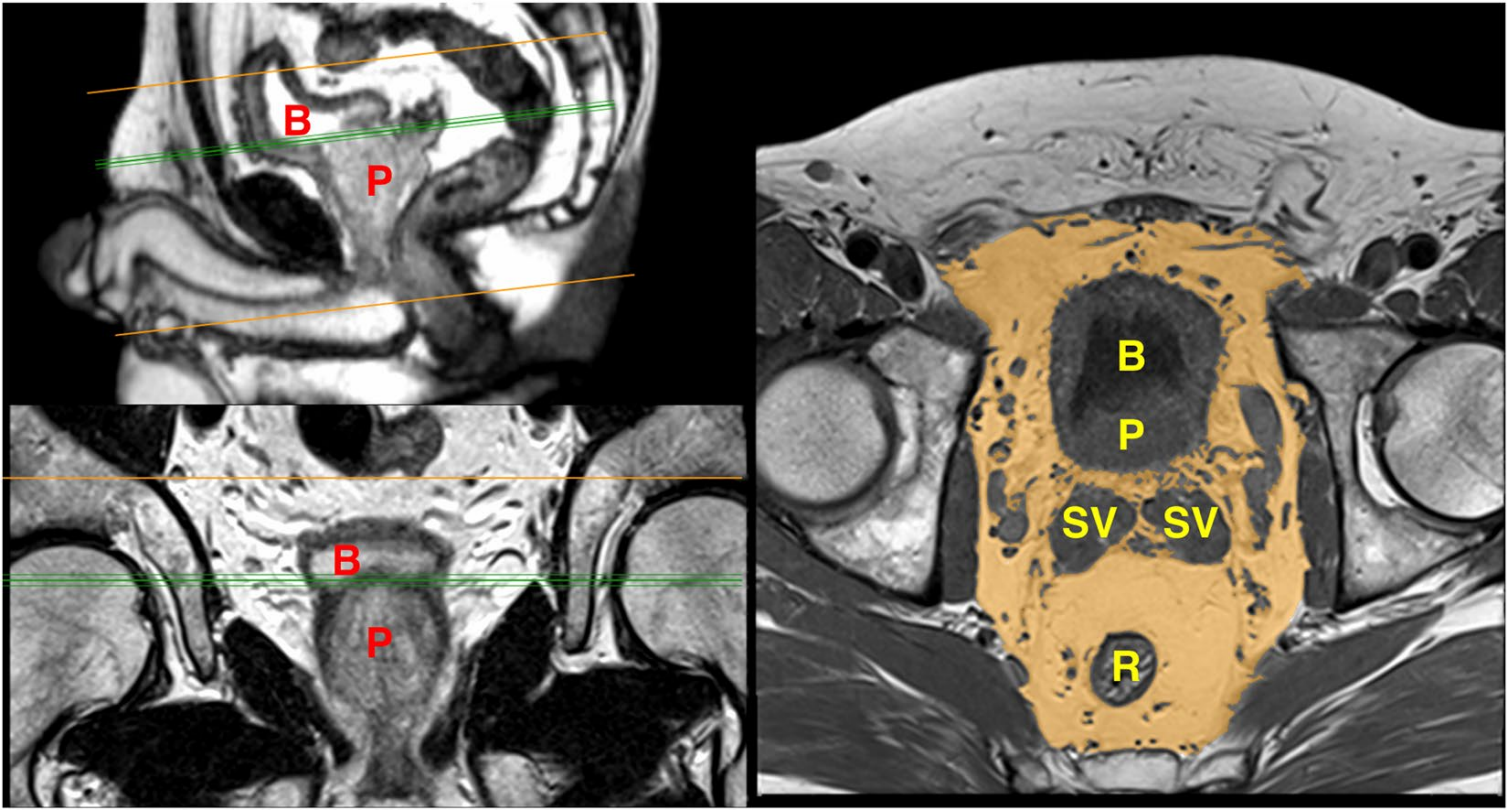
Sagittal and coronal images were used to identify the first slice at the level of the base of the prostate represented by the green lines. The yellow area represents the periprostatic fat, which was segmented from multiple sequential slices starting from this level to the prostate apex. P = Prostate gland; B = Bladder; R = Rectum; SV = Seminal Vesicle.

**Figure 3:**
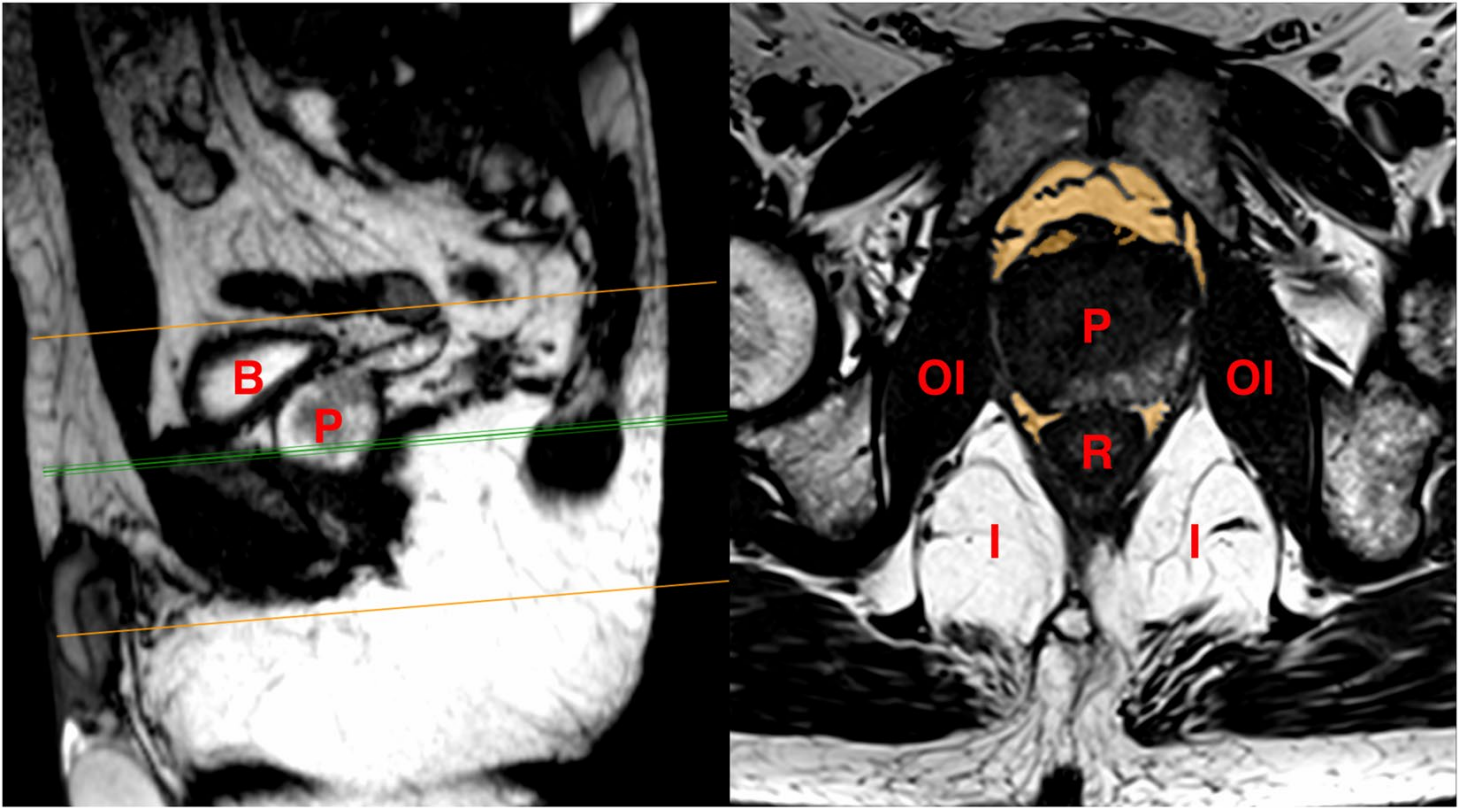
Segmentation technique to measure the volume of periprostatic fat (masked in yellow) from multiple sequential slices of axial T_1_ weighted MRIs. P = Prostate; B = Bladder; OI = Obturator internus muscle; R = Rectum; I = Ischiorectal fossa.

**Figure 4:**
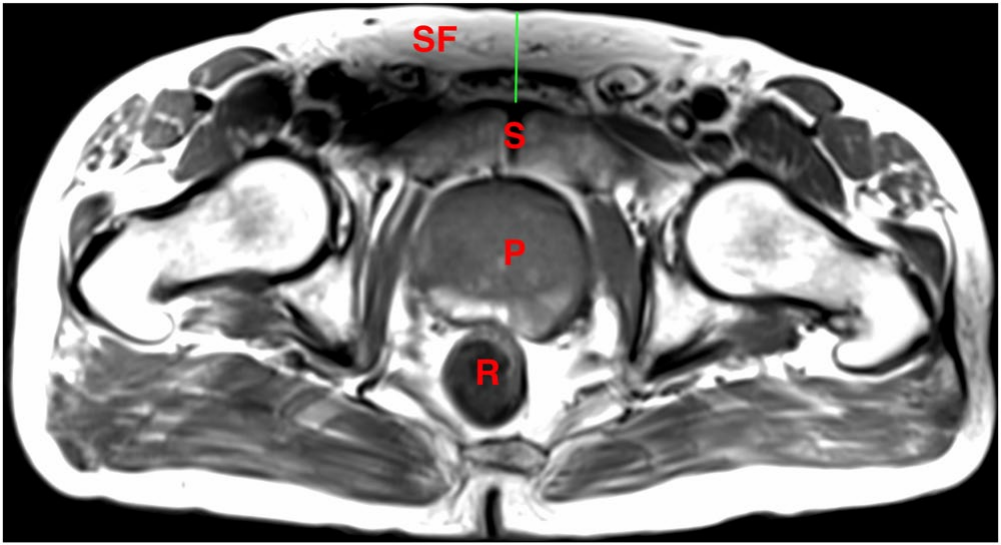
Measurement of subcutaneous fat thickness from the perpendicular distance between symphysis pubis and skin at the level of the superior pubic ramus on T_1_ weighted transverse pelvis MRI. P = Prostate gland; R = Rectum; S = Symphysis pubis; SF = Subcutaneous fat.

**Figure 5:**
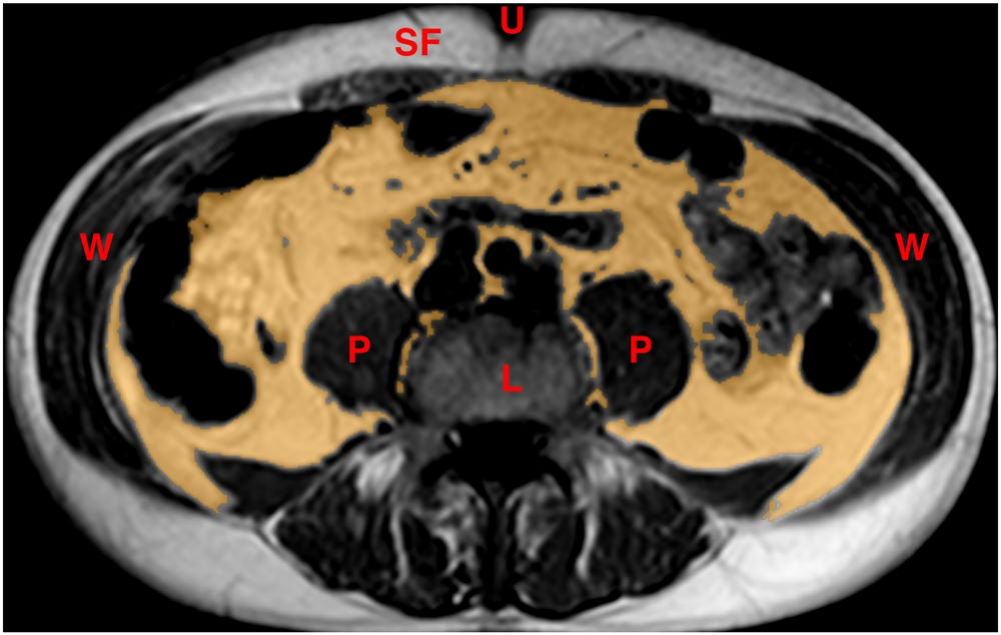
Abdominal fat area (masked in yellow) was measured from T_1_ weighted transverse abdominal MRI at the level of umbilicus using segmentation technique. L = Lumbar vertebral body; P = Psoas muscle; SF = Subcutaneous fat; U = Umbilicus; W = Abdominal wall muscles.

**Figure 6:**
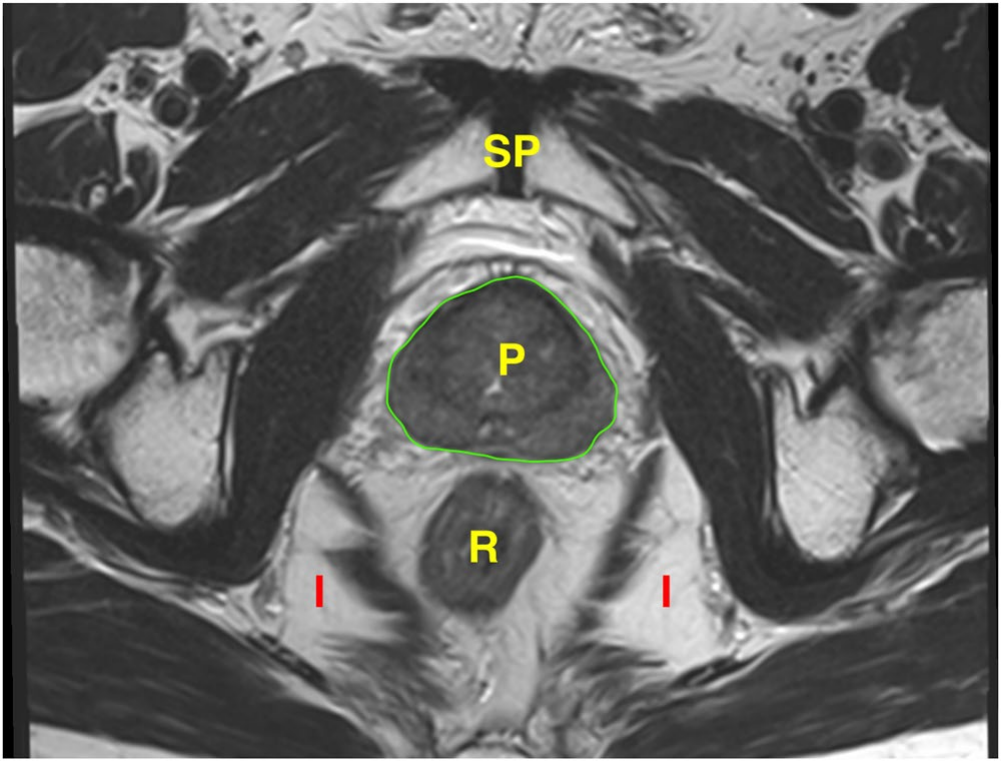
Prostate volume was measured from T_2_ weighted axial sequential images. The region of interest was marked manually from each slice using pencil starting from the level of the base of the prostate till the level of its apex. Subsequently the software automatically calculates the volume. P = Prostate gland; R = Rectum; I = Ischiorectal fossa; SP = Symphysis pubis.

The periprostatic fat was segmented from the level of the prostate base to the apex (Figs 2 and 3). The subcutaneous fat thickness (SFT) was determined by measuring the perpendicular distance between the skin and the anterior upper border of the symphysis pubis on a selected T1-weighted small field of view axial slice (Fig. 4). The abdominal fat area (AFA) was determined by segmenting the intra-abdominal fat on a single T1-weighted axial slice at the level of umbilicus including retroperitoneal fat (Fig. 5). Prostate volume (PV) was measured from multiple contiguous T2-weighted SFV axial images (Fig. 6). Since the dimensions of the prostate gland including the distance between apex and base are highly variable between individuals, PFV was normalised for all patients to account for the variations in PV (NPFV = PFV/PV). NPFV is the normalised value of periprostatic fat volume measured from the base of the prostate to its apex.

Reproducibility of PFV and PV measurements was assessed in a subgroup of 10 randomly selected study patients by three observers. An excellent interrater reliability was shown with intraclass correlation coefficient of 0.984 in single measures (*P* < 0.001).

### Statistical Analyses

Patients were stratified into three groups according to the Gleason score of the final prostatectomy specimen: ≤6, 7(3 + 4) and 7(4 + 3) or more. The association between the three groups and different fat measurements including clinical and pathological data was determined using one-way ANOVA and Kruskal-Wallis tests for parametric and non-parametric continuous variables, respectively, and Chi-square test for categorical variables (WHO weight classification, pT and D’Amico risk classification). Receiver Operating Characteristic (ROC) curve analysis was used to test the ability of NPFV to differentiate between high-grade (GS ≥ 7) and low-grade (GS ≤ 6) prostate cancers.

Pearson correlation coefficient was used to evaluate the relationship between the different fat measurements (NPFV, AFA and SFT), including BMI, and prostate cancer aggressiveness [GS: ≤6, 7 (3 + 4), 7 (4 + 3), 8 and 9, pT: pT_2_ and pT_3_]. After classifying the patients based on D’Amico risk classification, binary logistic regression analysis was used to determine an independent effect of each predictor on high-risk disease (biopsy GS ≥ 8 or PSA > 20, more than one positive core, or intermediate-risk and more than 50% positive cores) vs non-high risk (low and intermediate-risk) disease. *p* value of <0.05 was considered statistically significant. IBM SPSS Statistics (version 23) for OS X was used for data analyses.

## Results

The mean age of the cohort (*N* = 162) was 67.3 ± 5.7 years (range, 53–87), and the mean BMI was 27.6 ± 4 kg/m^2^ (range, 20.5–40.6). According to the WHO classification, 47 patients were classified as normal weight (30.1%), 70 as overweight (44.9%) and 39 as obese (25%). Table 3 summarizes the characteristics of patients categorised into three groups according to post-operative Gleason score of histopathology.

**Table 3:** Patient characteristics.

	Group 1 6	Group 2	Group 3	*p* value
	Post-operative Gleason score ≤	Post-operative Gleason score 7 (3 + 4)	Post-operative Gleason score 7 (4 + 3) and over	
Mean ± Standard Deviation				
No.	*n* = 25	*n* = 71	*n* = 66	
Age (years)	65.72 ± 6.07	66.45 ± 5.82	68.70 ± 5.07	0.022^a^
NPFV (cm^3^)	1.84 ± 0.76	2.45 ± 1.13	2.8 ± 1.56	0.001^a^
AFA (cm^2^)	137.51 ± 65.12	126.12 ± 50.75	144.42 ± 61.27	0.177^a^
SFT (cm)	3.84 ± 1.56	3.7 ± 1.18	3.8 ± 1.34	0.853^a^
BMI (kg/m^2^)	27.87 ± 4.69	27.31 ± 3.61	27.73 ± 4.28	0.781^a^
Initial PSA (ng/ml)	9.86 ± 5.29	11.06 ± 5.71	15.21 ± 11.11	0.006^b^
PV (cm^3^)	63.29 ± 26.99	54.71 ± 32.07	51.01 ± 19.94	0.051^b^
WHO classification *N* (%)*				
No.	*n* = 23	*n* = 69	*n* = 64	0.946^c^
Normal weight	7 (30.4)	22 (31.9)	18 (28.1)	
Overweight	9 (39.1)	31 (44.9)	30 (46.9)	
Obesity	7 (30.4)	16 (23.2)	16 (25)	
Pathological stage *N* (%)*				
No.	*n* = 25	*n* = 71	*n* = 66	0.001^c^
T_2_	20 (80)	43 (60.6)	25 (37.9)	
T_3_	5 (20)	28 (39.4)	41 (62.1)	
D’Amico risk classification *N* (%)*				
No.	*n* = 25	*n* = 71	*n* = 66	<0.001^c^
Low	18 (72)	18 (25.4)	2 (3)	
Intermediate	6 (24)	40 (56.3)	27 (40.9)	
High	1 (4)	13 (18.3)	37 (56.1)	

NPFV = Normalised periprostatic fat volume; AFA = Abdominal fat area; SFT = Subcutaneous fat thickness; BMI = Body mass index; PSA = Prostate specific antigen; PV = Prostate volume.

Patients were stratified according to post-operative Gleason score.

^a^ANOVA, ^b^Kruskal-Wallis test, ^c^ *χ* ^2^ test.

*(%) within each group.

*P* value is significant <0.05.

The mean age was statistically different between the three groups [F (2,159) = 3.9, *p* = 0.022], patients with more aggressive disease tending to be older. The mean NPFV was also statistically different between the groups [F (2,159) = 7, *p* = 0.001], and Tukey post-hoc test revealed that NPFV was statistically significantly larger in Group 2 (2.45 ± 1.13, *p* = 0.047) and Group 3 (2.79 ± 1.15, *p* = 0.001) than in Group 1 (1.84 ± 0.76). There was no statistically significant difference between Group 2 and Group 3 (*p* = 0.156). Patients with stage pT_2_ had statistically significantly lower NPFV (2.28 ± 0.98) than patients with stage pT_3_ (2.76 ± 1.25), t(160) = −2.760, *p* = 0.006.

Dichotomising PCa into low (GS ≤ 6) and high (GS ≥ 7) grade groups, ROC curve analysis yielded an area under the curve of 0.71, with Youden’s Index of 1.81 for a sensitivity of 73.7% and specificity 60% (*p* ≤ 0.001).

The NPFV was significantly correlated with post-operative GS (ρ = 0.294, *p* < 0.001). BMI was significantly positively correlated with AFA (ρ = 0.640, *p* < 0.001) and SFT (ρ = 0.794, *p* < 0.001). PSA was correlated with AFA (ρ = 0.207, *p* = 0.008) and SFT (ρ = 0.162, *p* = 0.039) but not to BMI (ρ = 0.121, *p* = 0.132) (Table 4). Neither BMI, AFA nor SFT correlated with post-operative GS (Table 4), nor was there any relationship with pathological stage (pT_2_ and pT_3_), [t (160) = 0.430, *p* = 0.668], [t(160) = 0.305, *p* = 0.760] and [t(154) = 0.492, *p* = 0.43], respectively. Twenty-six men had upgradation of GS from 6 to 7 and above from TRUS biopsy to final histopathology of the radical prostatectomy specimen. Mean NPFV of these men (2.43 ± 0.98) was more than those who had no upgrade from GS 6 (*n* = 17; 1.99 ± 0.82). This did not reach statistically significance, however, due to low numbers (*p* = 0.132).

**Table 4:** The relationship between post RP Gleason score, age, body mass index (BMI), prostate-specific antigen (PSA) and different fat measurements using Pearson correlation coefficient.

Correlations								
		NPFV	AFA	SFT	PSA	BMI	Biopsy GS	Post-op GS
Age	Pearson C.	0.154	0.046	−0.090	0.061	−0.149	0.133	0.147
	Sig. (2-tailed)	0.050	0.561	0.256	0.444	0.064	0.092	0.062
	*N*	162	162	162	162	156	162	162
NPFV	Pearson C.	1	0.129	0.042	0.036	0.117	0.331**	0.294**
	Sig. (2-tailed)		0.101	0.596	0.645	0.146	0.000	0.000
	*N*	162	162	162	162	156	162	162
AFA	Pearson C.	0.129	1	0.644**	0.207**	0.640**	0.098	0.046
	Sig. (2-tailed)	0.101		0.000	0.008	0.000	0.213	0.563
	*N*	162	162	162	162	156	162	162
SFT	Pearson C.	0.042	0.644**	1	0.162*	0.794**	0.062	−0.004
	Sig. (2-tailed)	0.596	0.000		0.039	0.000	0.435	0.960
	*N*	162	162	162	162	156	162	162
PSA	Pearson C.	0.036	0.207**	0.162*	1	0.121	0.169*	0.326**
	Sig. (2-tailed)	0.645	0.008	0.039		0.132	0.031	0.000
	*N*	162	162	162	162	156	162	162
BMI	Pearson C.	0.117	0.640**	0.794**	0.121	1	0.079	0.020
	Sig. (2-tailed)	0.146	0.000	0.000	0.132		0.326	0.800
	*N*	156	156	156	156	156	156	156

*Correlation is significant at the 0.05 level (2-tailed).

**Correlation is significant at the 0.01 level (2-tailed).

NPFV = Normalised periprostatic fat volume; AFA = Abdominal fat area; SFT = Subcutaneous fat thickness; PSA = Prostate specific antigen; BMI = Body mass index; GS = Gleason score.

Binary logistic regression analysis showed that NPFV was the only independent predictor of having high-risk PCa after controlling for age (OR, 1.398; 95% CI, 1.037–1.883; *p* = 0.028). Age was not an independent predictor of having high-risk PCa (OR, 0.969; 95% CI, 0.911–1.03; *p* = 0.313). Hosmer-Lemeshow test revealed that the data fitted the model well [***χ*** ^2^(8) = 5.273, *p* = 0.728].

## Discussion

This is the first prospective study that reports on the periprostatic and pelvic fat volume measured from contiguous T1-weighted axial images using a semi-automated segmentation technique, showing a strong correlation between prostate post-operative Gleason score and normalised periprostatic fat volume. In contrast to other studies conducted in the America and Canada^1–3, 18^, our observations show that periprostatic fat correlates better with GS than BMI, the marker of general obesity, which had no relation to PCa aggressiveness (as defined by GS) in this study. Our findings are, however in agreement with other reports from Europe suggesting no association between BMI and risk of being diagnosed with more aggressive prostate cancer^8, 19^. The discrepancy between the results needs further exploration, yet it could reflect differences in the percentage of the adult population who are clinically obese, which is higher in North America, and may be affected by the greater proportion of African Americans who tend to have more aggressive disease compared to Caucasian Americans^20–24^.

Recent studies have confirmed the relationship between periprostatic fat and PCa aggressiveness using different measurement techniques on trans-rectal ultrasonography (TRUS), CT and MRI^8, 15, 16, 25^. There are several differences between the previous studies and our study. Firstly, we measured periprostatic fat volume using a dedicated software rather than thickness of fat or area at a single point. Secondly, we have used RP as a reference standard, as it is established that there is a discrepancy between histological grade obtained during biopsies and final histopathology of prostatectomy specimen after radical surgery^26^, and confirmed in the results presented in this study.

Van Roermund *et al*.^8^ evaluated periprostatic fat as a predictive marker for prostate cancer aggressiveness using a single 3 mm-thick CT slice^8^. They showed that the periprostatic fat area and density (defined as periprostatic fat area/total contour area) (%), were predictors for cancer aggressiveness. Woo *et al*.^15^ reported that there was a positive correlation between periprostatic fat thickness, which was measured on a single preoperative mid-sagittal T1-w MRI from the symphysis pubis to the prostate, and GS^15^. Body fat distribution differs between individuals according to differences in age, angiogenesis, adipogenesis, genetic regulation and insulin resistance^27–29^. Moreover, since the prostate tumour and cancer-related adipocytes can be located at any level in the gland (most commonly in the posteriorly located peripheral zone), we considered that measuring the volume of periprostatic fat from the level of the prostate base to its apex was most appropriate.

There are several reports on the correlation between peritumoural fat and cancer outcomes. It has been suggested that retroperitoneal fat should be included in visceral fat measures as leptin, which promotes PCa progression, and adiponectin were associated with retroperitoneal fat area^30, 31^. Ohwaki *et al*.^32^ suggested that BMI is not an accurate marker for abdominal obesity assessment, and that the visceral (intraperitoneal) fat is the most accurate measurement for abdominal obesity^32^. Van Roermund *et al*.^7^ also concluded that the visceral fat could be more metabolically active than periprostatic fat after observing the relationship between BMI, SFT and periprostatic fat density^7^. Harada *et al*.^33^ measured the abdominal fat area at the level of umbilicus on 507 CT images, and concluded that low visceral fat contents were associated with poor prognosis in patients with upper gastrointestinal cancers^33^. In the present study, we measured AFA from a T_1_-weighted axial image at the level of the umbilicus including retroperitoneal fat. AFA and SFT were significantly positively correlated with BMI but not related to grade and stage of prostate cancer. Visceral fat, unlike periprostatic fat, could be related to upper gastrointestinal cancers but not to prostate cancer. This suggests that a close spatial relationship between a cancer and its peritumoural fat tissue is important in consideration of aggressivity and outcomes.

In this study, NPFV was positively correlated with both grade and stage of prostate cancer. A previous study showed that the adipocytes spatially related to a cancer release free fatty acids through lipolysis which are then used by the cancer cells for bioenergy^11^. Mitochondrial β-oxidation of the free fatty acids was found to be the dominant source of bioenergy for PCa cells^10^. This may indicate that both fat amount and activity have an impact on prostate cancer progression.

Anatomical MRI scans cannot distinguish between less or more active adipocytes of periprostatic fat tissue. Powell^34^ reported that there are two types of adipocytes, “fat” and “thin”^34^. The activated “fat” adipocytes are common in obese patients and produce more adipokines than “thin” adipocytes^34^. A few laboratory studies have concluded that there is a relationship between active periprostatic adipose tissue and prostate cancer cell survival, migration and higher risk of biochemical recurrence^10, 35^. Finley *et al*.^36^ investigated the association of adipose tissue cytokines with PCa aggressiveness^36^. IL-6 levels of both serum and periprostatic adipose tissue were compared with Gleason score (GS). The results showed that IL-6 secreted by periprostatic adipose tissue was approximately 375-fold greater than serum IL-6, and positively correlated with GS. They also mentioned that adipocytes of periprostatic fat tissue are the major secretors of IL-6 in patients with PCa. For future studies, it could be informative to further investigate both periprostatic fat adiposity and metabolic activity as the results may better reveal the relationship between the active periprostatic fat and PCa aggressiveness.

This study has some limitations. The slice-by-slice semi-automated segmentation technique was time consuming. Though the Osirix software factors in any interslice gap into volumetric calculations, a 3D volumetric acquisition with no interslice gap might allow more accurate segmentation. However, the theoretical impact of this on our results is likely to be minimal, since the same sequences were used in all subjects and slice thickness and gap were the same for the T_1_ and T_2_-weighted sequences used for determination of NPFV and PV, respectively. Furthermore, the segmentation technique showed excellent reproducibility. Though Dixon sequences are regarded as the gold standard in the determination of fat and water volumes, some form of user-defined thresholding is still necessary with semi-automated softwares. It could be argued that the sequences routinely used in 2010 when recruitment to this study commenced would not be regarded as ideal for multiparametric MRI characterisation of PCa according to PI-RADS v. 2 criteria. However, this will not have affected the research question addressed here; namely, the relationship of NPFV to PCa aggressivity.

A further limitation is that our inclusion criteria, using radical prostatectomy Gleason score as a reference standard, has restricted our cohort to 162 patients over a long period of time (2010–2015). Using this strict reference standard may limit the external validity of findings from the present study and introduce a selection bias, as patients with low Gleason score disease may not opt for radical surgery and may instead go onto active surveillance as a treatment choice. In the present study, there was a higher NPFV in men with upgradation of disease from Gleason 6 score on biopsy to GS > 6 following radical prostatectomy compared to those who continued to have low GS disease. The discriminatory power of NPFV in this clinical situation needs further study due to low number of cases. Even accepting the low numbers, we have demonstrated with ROC analysis that NPFV is clearly able to differentiate between high- and low-risk disease suggesting that it could be used to stratify patients into differing treatment groups. Observation from the present study and previous reports, however, do merit future research in selecting men for active surveillance using periprostatic fat volume as marker of aggressive disease, particularly in those where there is suspicion of poor sampling using ultrasound guided biopsies.

## Conclusions

In the present study, normalised periprostatic fat volume measured from MRI images significantly correlated with prostate cancer aggressiveness (grade and stage) in men who underwent radical prostatectomy for clinically non-metastatic disease. There was no correlation between BMI, AFA and SFT and prostate cancer aggressiveness. Defining the metabolic activity of periprostatic fat could add further to understanding the relationship between the active closely-related adipose tissue and PCa aggressiveness.
